# The History of the Horse

**Published:** 1881-04

**Authors:** Louis H. Laudy

**Affiliations:** Professor of Chemistry, Columbia Veterinary College


### THE JOURNAL
OF
### COMPARATIVE MEDICINE AND SURGERY.

VOL. II. APRIL, 1881. No. 2.

### ORIGINAL LECTURE.

### ART. VII.—THE HISTORY OF THE HORSE.

ADDRESS DELIVERED AT THE OPENING OF THE COLUMBIA VETERINARY COLLEGE AND SCHOOL OF COMPARATIVE MEDICINE, NEW YORK.

BY LOUIS H. LAUDY, PH. D.,
PROFESSOR OF CHEMISTRY, COLUMBIA VETERINARY COLLEGE.

WHEN we consider the large number of animals distributed over the whole earth's surface, and are called upon to recognize them individually, the task might well be considered as hopeless. But by a systematic arrangement of *classes*, *orders*, *genera*, and *species*, this can be accomplished with ease.

The first thing, therefore, to properly and fully understand is the *classification* of the immense series of living beings composing the animal creation. Whether the system adopted be that of *Owen*, *Cuvier*, or others, the leading features, which are the same, should be understood by all.

According to Cuvier, living animals are divided into Vertebrates, Mollusks, Articulates, Radiates.

The *Vertebrates* are those with a back bone, and include fishes, reptiles, birds, mammals.

The *Mollusks* form a very large sub-kingdom, and include animals of very various and irregular shapes, whose envelope is soft, but generally protected by a shell. Oysters, periwinkles, snails, cuttle-fish, etc. are examples of this sub-kingdom.

*Articulates* are animals enclosed in a jointed skin, as insects, spiders, lobsters.

*Radiates* include all the lowest and least perfect members ot the animal kingdom.*

With this classification of four divisions of animal life, it is possible to assign the animal to his proper position.

The completeness of the above classification, so far as it relates to the more perfect animals, is sufficiently great, and we may therefore, take it as our guide.

The grand character that distinguishes the *vertebrates* is that they are in possession of an internal jointed skeleton, which is endowed with vitality, nourished by blood-vessels, capable of growth, and which undergoes a perpetual renovation by the removal and replacement of the substances that enter into its composition. The complete skeleton of a *vetebrate* may be considered as being composed of several sets of bones, employed for different purposes, consisting of a central portion, the basis and support of the rest, and of various appendages derived from or connected with the central part. The nervous system of *vetebrates* consists of the *brain* contained within the cavity of the skull. Connected with this, and lodged in a canal formed by the back-bone is the *spinal cord* which connects with nerves through the entire body. With the increased development of the nervous system, the organs of the senses assume a proportionate perfection of structure. The *eyes*, two in number, are lodged in cavities in the bony framework of the face. The auditory apparatus gradually becomes more completely developed. Organs of *smell*, *taste*, and *touch*, are found fitted to exercise their different functions.

* To these four classes should be added a fifth, the Protozoa, which embraces the simplest forms of animal life. Another classification is the following: Vertebrata, Mollusca, Molluscoida Annulosa, Annuloida, Coelenterata, Protozoa.

The *blood* of all vertebrate animals is red, and contains microscopic corpuscles of variable form and dimensions. These are the leading characteristics that seperates them from all other orders. I have selected from the vertebrates one of the animals of the order, the *ungulata*, or hoofed quadrupeds, as this is the largest and most important of all the divisions of the mammalia.

It comprises three entire old orders, namely: *Pachyderms*, *Solipeds* and *Ruminants*.

The first of these old orders, *Pachyderms*, includes the elephant, rhinoceros, hippopotamus and others—the word Pachyderm meaning thick skin. The name is still used to express this fact, though the order is now abandoned. The second order includes the *Solipeds*, and in this order we find the horse, zebra and Ass. They are all characterized by the fact that the foot terminates in a single toe incased in an expanded hoof. They have a single stomach, and do not "chew the cud." The third order or *Ruminants* includes the *oxen*, *sheep*, *goats*, *camels*, *giraffes*, *deer*, and others that do chew the cud, or ruminate, and have two functional toes to each foot incased in hoofs. All these various animals are now grouped together into the single order of *Ungulata*, and the following are the characters of the order: All the four limbs are present, and that portion of the toe that touches the ground is always incased in a greatly expanded nail constituting a hoof. Owing to the incasement of the toes in hoofs, the limbs are useless for prehension, and only subserve locomotion, hence *clavicles* are wanting in the entire order. There are always two sets of enamelled teeth. The molar teeth are massive, and have broad crowns adapted for grinding vegetable substances.

The *Solipeds*, or animals of the genus *equus*, are those to which I wish now to call attention. The horse, is distinguished by the fact that the animal is not banded or striped, has no dorsal line, that both fore and hind legs have warts, and that the tail is covered with hair throughout. He is distinguished also by the fact that his hoof is undivided, and by the singular fact that he breathes through the nostrils only, and not through the mouth. Thus in outline I have given you the horse as Zoology teaches his position in the animal kingdom.

Let us pass from this and consider in brief the early history of the horse,which is a more notable one than that of any other animal. The announcement was that man should have dominion over every beast of the earth, yet the realization of that delegated power was the result of many centuries of slow and painful progress.

The history of Egypt furnishes, perhaps, the earliest record of the human family, and in it we find frequent record of the horse. By many it has been supposed that Egypt was his original home, while others dispute this, and claim that he was only distributed by the people of this ancient country. The country lying between the Tigris and the Euphrates, in Mesopotamia, where the whole race of mankind was collected together after the flood, this spot is given as the primitive home of the horse, and is the place where the best breeds (so far as the horse is concerned), are still found. This is the true Arabian horse. From this point he was easily brought into Egypt, where there was money to buy and sovereigns to use him.

In Biblical history the earliest notice we have of him occurs in Genesis, where the people are represented as giving Joseph in exchange for bread. The people mentioned in sacred history living upon the borders of Egypt, seemed to have placed a high value upon the *horse*, though all other domestic animals were abundant. Throughout India he continued to be very rare, even down to the days of Solomon, for that monarch obtained his horses from the land of Pharaoh.

Assyria, after Egypt, became celebrated for its cavaliers among the people of the ancient world. The prophets would exclaim of the horses of the Chaldeans, that "they are swifter than the leopards and more fierce than the evening wolves." Among the ruins of *Nimroud*, discovered by Layard, were found bas-reliefs representing Assyrians on horseback, not only engaged in hunting expeditions, but in actual war. Upon ruins in other cities of coeval date, horses and chariots are found in abundance, but in none is man seen mounted and going to battle. Until the discoveries of *Nimroud* it was supposed that the Persians originated equestrianism, but it seems that they only revived what had apparently been lost with the destruction of Ninevah. The plains Babylonia furnished horses to the Persians, which nation became renowned for its horsemanship. Persia, before the age of Cyrus, was nearly destitute of horses, but after that era, from personal example and encouragement of her kings, it is stated that every man in that vast empire rode on horseback. So rich did Persia become in equine wealth that one author speaks of no less than 150,000 horses feeding at one time upon a vast plain, near the *Capsian Gates.* The very name of the country comes from *Peresh,* a Hebrew word meaning *horseman.* We can thus trace through the dim traditions of the past, the horse leaving Egypt passing into Assyria and Persia, and following the rushing stream of population westward.

The Greeks soon became skillful horsemen, and they in turn taught the Romans their horsemanship, which nation soon rivaled its teachers. The Romans, it is said became more attached to their horses than did any other nation, and often carried the attachment to a height of folly and madness. Roman emperors have been known to invite their horses to sup with them, giving them food from golden vessels. They went to still greater extremes, making a horse high priest and consul. Verus erected a statute of gold to his horse, and fed him with raisins and almonds from his own hands. When the creature died he was buried with great pomp, all the dignitaries of the great empire attending, after which a magnificent monument was erected to his memory on the Vatican Hill. These two or three illustrations will give some idea of the horse's position, and the fondness displayed by owners at this early date.

The time and manner of the introduction of the Asiatic horse into Europe is not exactly known. The Persian war with the Greeks must have scattered them, as the Greeks captured at one time eighty thousand, many of which escaped. The Carthaginians introduced the Barbary horse into Spain and Sicily. From these two points the breed would naturally be dispersed over Western Europe. In the fourth century the Greeks who entered Europe by the north, and overran it as far as the Peninsula, dispersed the Asiatic horse in their track. In the eighth century the Saracens, with two hundred thousand horses, penetrated into France and were captured, and from this mixed breed was produced the Gallic horse, from which, no doubt, came the early English horses.

The introduction of horses upon the Western Continent dates back to the second voyage of Columbus in 1493. The first that were ever brought into the United States were landed in Florida in 1527.

These importations, from dissensions which followed among the masters, escaped all control, and finding a home in the wilderness, multiplied to an incredible extent. They, with the horses introduced into Mexico by Cortez, and into Peru by Pizarro, have filled the prairies of the north, and extended in countless numbers upon the pampas of the South. Upon the plains extending from La Plata to Patagonia are to be met single troops numbered by tens of thousands. The color of the "American wild horse," as some call it, is generally chestnut, but many are dark bay. They differ from those of Asia in being easily tamed. In fact, so readily are they subdued, that they cannot be said to exist in a truly savage state.

The speed of horses has always been a matter of admiration. The Orientals use the most beautiful expressions when alluding to this subject; they say, "Horses are birds which have no wings." "My steed has the wings of the wind." "Hast thou made him to leap forth like the arrow." Among the more matter-of-fact people of temperate climates, we find equal exaggeration, but expressed in less poetical language. The meaning of "low down in the twenties," is, if analyzed, quite as much as to say, for horses, nothing is distant.

Careful investigation clearly shows that *horses do not degenerate in America*, as some writers would try to prove. We have rather the best of the argument, for our *trotters* are the best in the world, as well as the most enduring, and when tried upon English ground have always maintained their reputation. No horse but an American one ever trotted *in harness* in 2-10¾. Before the introduction of railways, the horse was the swiftest mode of conveyance man possessed. When extraordinary distances were made in a given space of time, the details were sent around as exciting news, and recorded among the wonderful events of the day. Many cases could be cited, and all are full of adventure. Sir Robert Carey rode 300 miles in less than three days when he went from London to Edinburgh to inform King James of Queen Elizabeth's death. In more modern times General Lafayette displayed his zeal and strength by riding from Rhode Island to Boston, nearly seventy miles in seven hours, and delivering a message to Washington. He then immediately returned, making the distance in six-and-a-half hours.

A highly interesting volume might be written upon the *garniture of the horse.* It was not customary in ancient times to shoe his feet with iron according to our modern practice. In oriental countries, the dryness of the soil made an artificial defence of the hoof, less necessary than it is in the mire and the muddy ways peculiar to the north of Europe. Necessity first suggested the shoeing of horses and custom confirmed the practice. Yet nothing is more abused for a want of proper knowledge, than the imperfect methods of shoeing a horse as practiced by most of our horseshoers of the present day. As veterinary science advances each year, we find a corresponding inprovement in the care and treatment of horses feet, and with a full and proper knowledge of the anotomy of the foot, all the old methods will be superceded by those of good common sense horseshoeing.

The ancients used no saddles, judging from all sculptures that have been preserved. Many rode without even a bridal, and thus resemble the Indian tribes of our day. The sculptures of Khorsabad represent the riders "bare-back." The Elgin marbles are without saddles, but the want of these things is more than compensated by other trappings, in particular the bridal and reins which were of extraordinary splendor. In the bas-reliefs found in Nineveh, the trappings of the horses and chariots are remarkable for their richness and elegance. Above the heads rise gracefully plumed crests ornamented with long streamers of many colors. The bridal consists of a headstall, a strap divided into three parts, joining the bit together, with straps over the head, under the cheeks, and behind the ears.

The bits as well as the metal used in the harness were often of gold and other precious metals. The manes and tails were bound in the centre with ribbons adorned with tassels, and the cloakings were stndded with diamonds and precious stones. The most gorgeous display of cavalry of modern times must be incomparably behind those early days in everything that constitutes grandeur, either in numbers or costly equipments.

*The Horse as an Article of Diet.*—The size of the brute rendered it conspicuous, and it is presumed that he was first hunted alone for food. Compared with the flesh of the horse, the ox is coarse in the extreme. The muscular fibres of the horse are delicate, and the colors they displayed defy the pencil by their beauty. The muscles are arranged more symmetrically than those of any other animal, and the general aspect of the creature's frame, upon dissection, enforces the idea of a beast of superior order. Few have ever made themselves acquainted with the beautiful mechanical construction of the horse without imbibing a higher respect for him than is realized by the superficial examination of his outside form. But all this rather destroys than tempts the human appetite. Acceptable as the flesh may have been to the pallates of the early representatives of mankind, it is probable they were not able to indulge in an excess of this food, from the fact that the value debarred them, together with the difficulty of capturing the animal, as the horse cannot be taken by surprise. Once alarmed, pursuit was hopeless, and it is therefore probable that they were captured by means of pitfalls and other contrivances peculiar to the nation. The carcass was alone desired—the life of the victim was in no way regarded. The Scandinavians and Germans devoted to the worship of Odin, raised with the utmost care, in "sacred pastures," a breed of white horses that were sacrificed on certain occasions, and the flesh served up at the festive board From this perhaps originated the taste for such kind of food, a taste which existed among all the natious of the North, or until

Christianity penetrated Europe and succeeded in destroying this custom. The tribes of Northern Asia have preserved a marvelous liking for horseflesh. It is their favorite dish, although they have cattle in abundance.

Hazzard, a French veterinary surgeon, has stated that during the French Revolution, the greater portion of the meat consumed in Paris for six months was supplied by horses, and the public health did not suffer in consequence. In South America they esteem the horse the best of food, and prefer it to the wild cattle. The Danes, as late as the year 1807, during the siege of Copenhagen, authorized the sale of this meat.

Belgium may be quoted as having partially followed the example of Copenhagen, and the Austrian Government has recently authorized the sale of horseflesh. In Sweden horseflesh is looked upon as a great delicacy, and the wealthy class take a sandwich composed of bread and a thin slice of the animal highly salted, and this is considered a great treat. In this country most people are shocked at these practices, and will turn away disgusted with the offer to partake of what in other countries is considered a great treat. At first mention we heartily condemn the custom, yet we eat our bologna sausages without a wry face, and have thus been initiating ourself into the mysteries of consuming horse meat.

The *age of the horse*, up to eight or ten years, is correctly given by an examination of the teeth, those useful appendages through that period of time undergoing more transformations than in any other animal. It requires the practiced eye of a veterinarian to determine with a degree of precision the age of a horse, although we find many that will examine the mouth from mere habit, and then turn aside with a knowing look or a sly wink, and whisper, "twelve years." Should you ask them how they determine the age, they will tell you that their grandfather posted them, and that no horse doctor could cheat them.

When *well* treated, the animal lives to a good old age, but from the abuses he receives, his best years are from five to ten. Oftentimes he will remain serviceable for twenty years or more. An incident is recorded of a horse reaching the wonderful age of seventy years. "Copenhagen," the charger that carried Wellington at Waterloo, died at the age of twenty-seven. Copenhagen was buried with military honors, but his remains failed to remain long in repose; a curiosity hunter dug them up, stole one of the hoofs and a few other parts, and made good his escape without ever being discovered. The skeleton of "Marengo," the horse Napoleon rode at Waterloo, graces the museum of the United Service at London. This is in a glass case, and there is no chance to get a hoof.

*Price of the Horse.*—In all ages horses have commanded high prices. In Solomon's time great prices were paid for horses. The wholesale price under government contract was 150 shekels, which is about eighty-seven dollars, a great sum of money in those days. To form some idea of his wealth in horses, I will present a few figures. Solomon had four hundred stables, forty thousand stalls, and twelve thousand horsemen, an amount of money invested that, even in our extravagant days, would figure in the millions.

In our own times we find enormous sums paid for horses, and price bears a certain ratio to the speed.

As the time is reduced, so is the horse's value increased; and it might be something in this order:

| | | |
|---|---|---|
| Time | 2.10, not reached as yet. | |
| " | 2.10¼ | $175,000. |
| " | 2.12 | 100,000. |
| " | 2.15 | 75,000. |
| " | 2.25 | 5,000. |
| " | 2.35 | 1,000. |

From this to three or five minutes, the price is according to style and action, until at last it is a question whether the horse pulls you or you pull the horse.

*Diseases of the Horse.*—The constitution of no animal seems to be more subject to derangement and disease than the horse. For the alleviation of these and other suffering animals, the Columbia Veterinary College has been established. The student of veterinary science should be deeply impressed with the importance, nay, the indispensable necessity, of a profound and intimate knowledge of the entire structure of the horse. Such knowledge can only be obtained by a systematic course of study. Although veterinary science is becoming more prominent in this country, its importance is not as yet appreciated as it should be, for it is only within the past few years that it has been accepted as an educational branch of study.

By slow degrees and perseverance, with the valuable aid of many well-trained minds, there has been finally established a well-arranged system of teaching veterinary medicine. We have also now many excellent text-books on the subject of the anatomy and pathology of the domestic animals.

*Columbia Veterinary College.*—With such helps we have established a college that has met with an assured success, and which stands as a monument to attest our wisdom in our undertaking.

The intelligent citizens of this community cannot fail to appreciate the liberality of the many minds that have worked together to build up this institution. We have labored together and have systematized a course of studies that are comprehensive and as nearly complete as possible, and important to the wants of the veterinary student. This institution has been established at private expense. Time, talents and money have alike been devoted to promoting its worth and success. It is hoped that the institution will be found every way worthy of the cause to which it is devoted.

